# Prevalence and factors associated with human brucellosis in livestock professionals

**DOI:** 10.1590/S1518-8787.2017051006051

**Published:** 2017-06-13

**Authors:** Franco Cazembe Mufinda, Fernando Boinas, Carla Nunes

**Affiliations:** I Escola Nacional de Saúde Pública. Universidade Nova de Lisboa. Lisboa, Portugal; II Direcção Provincial de Saúde do Namibe-Angola. Moçâmedes, Namibe, Angola; III Centro Interdisciplinar de Sanidade Animal. Faculdade de Medicina Veterinária. Universidade de Lisboa. Lisboa, Portugal; IV Centro de Investigação em Saúde Pública. Universidade Nova de Lisboa. Lisboa, Portugal

**Keywords:** Brucellosis, epidemiology, Animal Husbandry, Occupational Risks, Socioeconomic Factors, Seroepidemiologic Studies

## Abstract

**OBJECTIVE:**

The objective of this study is to estimate the seroprevalence of human brucellosis in livestock professionals and analyze the factors associated with brucellosis focusing on sociodemographic variables and the variables of knowledge and practices related to the characteristics of the activities carried out in livestock.

**METHODS:**

This is a cross-sectional seroepidemiological study with a population of 131 workers of butchers, slaughter rooms, and slaughterhouse and 192 breeders sampled randomly in Namibe province, Angola. The data were obtained from the collection of blood and use of questionnaires. The laboratory tests used were rose bengal and slow agglutination. The questionnaire allowed us to collect sociodemographic information and, specifically on brucellosis, it incorporated questions about knowledge, attitudes, and behaviors of livestock professionals. In addition to the descriptive statistical approach, we used the Chi-square test of independence, Fisher’s test, and logistic regression models, using a significance level of 10%.

**RESULTS:**

The general weighted prevalence of brucellosis was 15.6% (95%CI 13.61–17.50), being it 5.3% in workers and 16.7% (95%CI 11.39–21.93) in breeders. The statistical significance was observed between human seroprevalence and category (worker and breeder) (p < 0.001) and education level (p = 0.032), start of activity (p = 0079), and service location (p = 0.055). In a multivariate context, the positive factor associated with brucellosis in professionals was the professional category (OR = 3.54; 95%CI 1.57–8.30, related to breeders in relation to workers).

**CONCLUSIONS:**

Human brucellosis in livestock professionals is prevalent in Namibe province (15.6%), where the professional category was the most important factor. The seroprevalence levels detected are high when compared with those found in similar studies.

## RESUMO

**OBJECTIVO:**

Estimar a seroprevalência da brucelose humana em profissionais da pecuária e analisar os factores associados à brucelose com foco em variáveis sociodemográficas, de conhecimento e práticas relativas às características das actividades desenvolvidas na pecuária.

**MÉTODOS:**

Estudo transversal seroepidemiológico em população de 131 trabalhadores de talhos, salas de abate e matadouro e 192 criadores amostrados aleatoriamente na província do Namibe, Angola. Os dados foram obtidos por meio de colheita de sangue e aplicação de questionário. Os testes laboratoriais utilizados foram o Rosa de Bengala e a aglutinação lenta em tubos. O questionário permitiu recolher informação sociodemográfica e, especificamente sobre a brucelose, incorporou questões sobre conhecimento, atitudes e comportamentos dos profissionais da pecuária. Além da abordagem estatística descritiva, foram utilizados os testes de Independência do Qui-quadrado, Fisher e modelos de regressão logística, utilizando um nível de significância de 10%.

**RESULTADOS:**

A prevalência geral ponderada da brucelose foi de 15.6% (IC95% 13.61–17.50), sendo 5.3% em trabalhadores e 16.7% (IC95% 11.39–21.93) em criadores. A significância estatística foi observada entre a seroprevalência humana e a categoria (trabalhador e criador) (p < 0.001) e o nível de instrução (p = 0.032), início de actividade (p = 0.079) e local de serviço (p = 0.055). Em um contexto multivariado, o factor positivamente associado à brucelose em profissionais foi a categoria profissional (OR = 3.54; IC95% 1.57–8.30, relativo aos criadores em relação a trabalhadores).

**CONCLUSÕES:**

A brucelose humana em profissionais da pecuária é prevalente na província do Namibe (15.6%), onde a categoria profissional foi o factor mais relevante. Os níveis de seroprevalência detectados são elevados se comparados com outros encontrados em estudos semelhantes.

## INTRODUCTION

Brucellosis is a zoonosis that comes from the direct or indirect contact with animal infection. It is an infectious disease caused by bacteria of the genus *Brucella*. In cattle, it is commonly caused by *Brucella abortus* ^9,20,30^.

Brucellosis is still a global problem of public health, with approximately 500,000 new cases of human infection every year, considering all the species of *Brucella*, although most developed countries have already controlled it^21,a,b^. In humans, it is manifested by fever and muscle and bone pain, and it is under-diagnosed worldwide^8^. In tropical countries such as Angola, where communicable infectious diseases are prevalent, brucellosis is confused symptomatically with several diseases, such as malaria, leptospirosis, and typhoid fever^b,c,d^. The literature reports that the most common causes of infection have been the labor conditions linked to exposed livestock professionals (veterinarians, slaughterhouse workers, and animal breeders) and consumption of infected products (meat, milk, and dairy products)^1,10^. The incidence of human infection varies depending on the degree of prevalence of animal infection, socioeconomic level, and eating habits^22^.

Ingestion, direct contact, and inhalation are indicated as the main forms of infection transmission, but the relative importance of the mode of transmission and gateways of the etiological agent vary according to the epidemiological area, animal reservoirs, occupational groups, and consumers exposed to the risk^8,10,13,c,d,e^.

The control of brucellosis involves animal disease eradication, control of the movement of unpasteurized milk and dairy products, compliance with biosecurity measures in the workplace of professionals at risk of infection (use of personal and collective protective equipment), and implementation of epidemiological surveillance for early detection of cases. These measures aim to establish barriers against the modes of contamination^5,10,e,f^.

The objective of this study was to estimate the seroprevalence of human brucellosis in livestock professionals in Namibe province, Angola, and analyze the factors associated with brucellosis focusing on sociodemographic variables and the variables of knowledge and practices related to the characteristics of the activities carried out in livestock.

## METHODS

This cross-sectional epidemiological study, conducted in 2012, took place in Namibe, one of the 18 provinces of the Republic of Angola (in the Southwest). Namibe has a surface of 57,097 km^2^ and an Atlantic maritime boundary line of approximately 480 km. Administratively, it consists of five municipalities: Namibe, Tômbwa, Virei, Kamucuio, and Bibala. Population is estimated at 1,195,779 inhabitants with a density of 21 inhab/km^2^, and its economy focuses mainly on fishing, cattle breeding, and agriculture^g^.

On the Northwest and Southeast of this province, the creation of all types of livestock is practiced, based on traditional and rudimentary techniques of veterinary care. The region has a herd of 500,500 heads of cattle^g^.

This study focused on livestock professionals, specifically cattle breeders and workers of slaughterhouse, butchers, and municipal slaughter rooms. Both work daily with animals and present increased risk of infection as they do not use biosecurity measures and consume unpasteurized milk and dairy products. Additionally, breeders have increased risks of contact with infected remains of abortions or postpartum^8,14,h^.

In December 2011, 131 workers were officially registered in the Provincial Department of Animal Husbandry of Namibe. The number of breeders was 1,204, distributed as follows in the five municipalities that make up Namibe: 748 (Bibala), 276 (Kamucuio), 118 (Virei), 51 (Namibe), and 11 (Tômbwa). All workers (131) were part of the study. In relation to breeders, we used a stratified random sampling, in which the strata proportionally represent the different municipalities^6^.

For an expected prevalence of human brucellosis of 5%, a margin of error of 3%, and a 10% increase to mitigate non-response or incomplete response, we set sample size at 192^i^. Thus, the respective sample sizes by strata (municipalities) were: Bibala (*n*_B_ = 113), Kamucuio (*n*_K_ = 44), Virei (*n*_V_ = 19), Namibe (*n*_N_ = 9), and Tômbwa (*n*_T_ = 7). The selection of breeders was carried out using a table of random numbers generated by the program OpenEpi®, version 2.3.1^j^.

For the serological study, we collected 5 ml of venous blood from each professional, with a G21 needle and 5 ml plastic syringe. Blood was centrifuged for subsequent removal of the serum. Serum samples were stored in plastic microtubes and frozen at -20ºC until serological testing.

Collections were carried out in situ, at health centers, or municipal hospitals. Serological tests were carried out in the Veterinary Research Institute of Huila and the Provincial Hospital Laboratory Ngola Kimbanda of Namibe.

The tests were done using a serial testing protocol starting with screening by the rose bengal test (RBT), and then the positive results were confirmed by the slow agglutination test (SAT)^4,e^, a common practice in the serological diagnosis of human brucellosis^1,2,e,k^.The seroprevalence result from the rose bengal test is classified as positive when the agglutination test is positive. The seroprevalence result from the SAT is considered as positive when it presents partial or complete agglutination. For identification of positive cases in this study, we set the cutoff point of SAT serology at 1:160^2,9^ with prior RBT^k^.

In 2009, based on a literature review, a questionnaire was developed on the knowledge, attitudes, and behaviors of livestock professionals, later translated by a traditional leader with a background in health to the local dialect (Nhaneca-Umbi)^b,c^. The same questionnaire was used herein, applied by local health agents, with local dialect proficiency and previously trained. This training consisted of basic information about the disease and application and filling of the questionnaire, and we clearly stated that their intervention could only clarify any questions about the content and interpretation of the questionnaire, and they could not influence the answer.

As for demographic characterization of the professionals under analysis, we considered the following variables: age group, gender, place of birth (province of Namibe and other), education level (no education and basic education), service location (SOFRIO Slaughterhouse and butchers of Namibe, municipal slaughter rooms, and farms of the different municipalities), start of activities (minor: < 18 years and adult: ≥ 18 years), and formal entry in the activity (legacy [heir] , entrepreneur, and hired).

Risk factors under analysis were knowledge of professionals and their practices: 1) have ever heard of brucellosis; 2) fresh milk transmits brucellosis; 3) animal fetal materials transmit brucellosis; 4) pasture is near water sources; 5) existence of wetlands along the pasture; 6) there is replacement of herd with cattle from other pens; 7) sale of fresh milk and unpasteurized dairy products; and 8) abortion remains are abandoned in the pasture. Questions 1, 2, and 3, which address knowledge, were made to all professionals (breeders and workers) and the rest (about the characteristics of the farm and practices), only to breeders. Except for the first question, “have ever heard of brucellosis”, which only allowed answering yes or no, the remaining questions had three response options (yes; no; does not know or no response).

For statistical analysis, we used the PASW Statistics 18.0®^l^. We considered a significance level of 10%.

After the descriptive approach, we used the Chi-square test of independence to analyze the relationship of prevalence with the sociodemographic variables and variables of knowledge and characteristics of farms. When the conditions of applicability of this test were not met, we used the Chi-square test of independence with Monte Carlo simulation or Fisher’s exact test. To identify the factors associated with seroprevalence (having brucellosis as the dependent variable) in professionals, we used logistic regression models with forward selection based on Wald test. This test is a stepwise method, in which the input of an independent variable in the model is done according to the score statistical significance, and the removal of a variable from the model is done according to the significance of the test to be defined. In this way, the initial model is saturated with the inclusion of all the explanatory variables (sociodemographic variables); in the various stages of the model, the variables that have less power of explanation of the prevalence variable are removed one by one, according to the significance of the test in use. The crude odds ratios were determined using the enter method with one explanatory variable at a time^14^. We also determined the confidence intervals for significant relationships. The odds ratio adjusted for gender and age are not presented as they were very similar to the crude odds ratio, bringing no additional information. We did not identify any multiple model, as only one of the variables was identified in the multiple analysis.

The apparent prevalence in the professionals was calculated by dividing the cases positive for the SAT by the total number of professionals. The same principle was applied to determine the prevalence in breeders and workers.

To calculate global prevalence (weighted prevalence), the prevalence by group of professionals was weighted considering the respective weights in the population under study, being 1,204 (90.2%) breeders and 131 (9.8%) workers in a population of 1,355 livestock professionals. The same logic was applied to determine prevalence in the municipalities.

We respected the guidelines of Helsinki and CIOMS-2002 (Council for International Organizations of Medical Sciences) regarding research with humans, avoiding any type of physical or moral damage^26,m^. The positive cases of brucellosis were subsequently referred to the State health units of Namibe and monitored free of charge. The study was approved by the Ethics Committee of the National Institute of Public Health of the Ministry of Health of the Republic of Angola. Informed consent was obtained from all participants. In the case of minors, we obtained the consent from their guardians.

## RESULTS

Of the total professionals assessed, 12.1% (39) showed positive result for brucellosis in both RBT and SAT (Table 1).

**Table 1:** Results of the RBT and SAT in humans, applied in series. Namibe, Angola, 2012.

Test	Negative		Positive		Total analyzed
	
	n	%	n	%	
RBT	279	86.4	44	13.6	323
SAT*	5	87.9	39	12.1	44

RBT: Rose Bengal Test; SAT: Slow Agglutination Test

* From the application in series of the test; the negative results of the RBT and the overall size of the sample (323) are considered when calculating the percentages of the SAT.

The general weighted prevalence of brucellosis was 15.6%, being it 5.3% in workers and 16.7% in breeders (p < 0.001). The municipality of Tombwa had the highest prevalence rate of infection in breeders (28.6%) and total professionals (26.9%). The municipality of Kamucuio had the highest prevalence rate of infection in workers (20%) (Table 2).

**Table 2:** Prevalence of human brucellosis. Namibe, Angola, 2012.

Municipality	Workers			Breeders			Total
		
	NT	TP	PT (%)	NC	CP	PC (%)	PP (%)
Namibe	103	4	3.9	9	1	11.1	10.4
Tombwa	9	1	11.1	7	2	28.6	26.9
Bibala	8	1	12.5	113	19	16.8	16.4
Kamucuio	5	1	20.0	44	6	13.6	14.3
Virei	6	0	0	19	4	21.0	19.0

Total	131	7	5.3	192	32	16.7	15.6

NT: population of workers; NC: sample of breeders; CP: positive breeders; TP: positive workers; PC: prevalence rate in breeders; PT: prevalence rate in workers; PP: weighted prevalence rates

The population examined consisted of 323 professionals, being most males and average age of 36.19 years, being the minimum 16, the maximum 71, and the standard deviation 13.23 years.

Of the 131 workers of slaughterhouse, municipal slaughter rooms, and butchers, 64.9% were males and 35.1% females. They had an average age of 33.27 years, with minimum of 17, maximum of 66, and standard deviation of 10.75 years.

Of the 192 cattle breeders, 84.9% were males and 15.1% females, average age was 38.18 years, with minimum of 16, maximum of 71, and standard deviation of 14.38 years (Table 3).

**Table 3:** Relationship of the seroprevalence of human brucellosis with sociodemographic variables of the professionals.

Variable	Total		Human seroprevalence				p

			Positive		Negative		
		
	n	%	n	%	n	%	
Category	323	100					0.001^a^
Worker	131	40.6	7	5.3	124	94.7	
Breeder	192	59.4	32	16.7	160	83.3	
Gender	323	100					0.703^a^
Male	248	76.8	29	11.7	219	88.3	
Female	75	23.2	10	13.3	65	86.7	
Age group (years)	323	100					0.469^b^
10–19	25	7.7	5	20.0	20	80.0	
20–29	102	31.6	14	13.7	88	86.3	
30–39	79	24.5	5	6.3	74	93.7	
40–49	55	17.1	8	14.5	47	85.5	
50–59	46	14.2	5	10.9	41	89.1	
> 60	16	4.9	2	12.5	14	87.5	
Place of birth	323	100					0.785^b^
Namibe province	209	64.7	26	12.4	183	87.6	
Other	114	35.3	13	11.4	101	88.6	
Education level	323	100					0.032^b^
No education	189	58.5	29	15.3	160	84.7	
Basic education	134	41.5	10	7.5	124	92.5	
Start of activities	323	100					0.079^b^
Minor	226	70.0	32	14.2	194	85.8	
Adult	97	30.0	7	7.2	90	92.8	
Formal entry in the activity	323	100					0.103^b^
Legacy (heir)	116	35.9	20	17.2	96	82.8	
Entrepreneur	109	33.7	10	9.2	99	90.8	
Contract	98	30.4	9	9.2	89	90.8	
Service location	323	100.					0.055^c^
Slaughterhouse SOFRIO and butchers of Namibe	103	31.9	4	3.9	99	96.1	
Municipal slaughter rooms	28	8.7	3	10.7	25	89.3	
Farms of Namibe	9	2.7	1	11.1	8	88.9	
Farms of Tombwa	7	2.2	2	28.6	5	71.4	
Farms of Bibala	113	35.0	19	16.8	94	83.2	
Farms of Kamucuio	44	13.6	6	13.6	38	86.4	
Farms of Virei	19	5.9	4	21.1	15	78.9	

^a^ Fisher’s exact test.

^b^ Chi-square test of independence.

^c^ Chi-square test of independence with Monte Carlo Simulation.

The relationships between seroprevalence of brucellosis and sociodemographic variables of the professionals are identified in Table 3, and their characterizations, for gender, age, and category and education level (both identified as significant) can be found in Table 4.

**Table 4:** Risk factors for human brucellosis in livestock professionals. Namibe, Angola, 2012.

Variable	Crude OR	95%CI	p
Category			
Worker*			
Breeder	3.54	1.57–8.30	0.004
Gender			
Male*			
Female	0.86	0.39–1.86	0.703
Age group (years)			
> 60*			
10–19	0.64	0.21–1.97	0.433
20–29	0.27	0.07–1.03	0.055
30–39	0.68	0.19–2.33	0.541
40–49	0.49	0.13–1.89	0.297
50–59	0.57	0.09–3.38	0.537
Education level			
Basic education*			
No education	2.25	1.06–4.79	0.036

* Reference class.

Of the total infected professionals (39), 82.1% were breeders and 17.9% were workers of slaughterhouse, butchers, and municipal slaughter rooms. The difference between the two groups was statistically significant (p < 0.001). The percentage of seropositive workers and breeders was 5.3% and 16.7%, respectively (OR = 3.71).

The age group of 20–29 years had 35.9% (14/39) of the infected professionals. In the age group of 10–19 years, the infected professionals accounted for 20% (5/25), and in the age group of 30–39 years they were 6.3% (5/79), the latter being the lowest value observed. Of the total of those infected (39), 76.9% (30/39) were married and 23.1% (9/39) were single.

Of those infected, 66.7% were from Namibe. In the group of those from Namibe, 12.4% were positive. In those from other provinces, 11.4% were positive.

Of those infected, 74.4% were professionals without education. In the group of illiterate persons, 15.3% were positive, and of those who had basic education, 7.5% were positive, with an OR of 2.25. Of the infected professionals, 82% started their activity as minors. Of the professionals who are in this group, 14.2% were positive, and in the group of those who started their activity in adulthood, 7.2% were positive.

Approximately 51.3% of the infected persons started the activity from the inheritance of animals, 25.6% from entrepreneurship, and 23.1% from contract. In the group of those who inherited the activity, 17.2% were positive; of those who were entrepreneurs, 9.2% were positive, and of those hired, 9.2% were positive (p = 0.103).

Of those infected, 48.7% of the breeders were from the municipality of Bibala, and a tenth (10.2%) of the professionals were slaughterhouse workers. In the farms of the city of Tombwa, the infection represented 28.6% (2/7); while in the SOFRIO slaughterhouse and butchers of Namibe, it was 3.9% (4/103) (p = 0.055).

The relationship between the seroprevalence of human brucellosis and the knowledge of risk factors, characteristics, and practices of farms are described in Table 5. Of the infected professionals, 15.4% claimed to have heard of brucellosis, with no relationship between being infected and having heard of the disease (p = 0.411), 33.3% considered that fresh milk transmits brucellosis, and 66.7% did not know or did not reply (p = 0.704). Additionally, 83.3% said that animal fetal materials do not transmit brucellosis (p = 0.633). In relation to both factors mentioned, ignorance, considering the does not know or no answer and does not transmit, is more prevalent in those not infected.

**Table 5:** Relationship of the seroprevalence of human brucellosis with knowledge and prophylaxis in professionals.

Knowledge and prophylaxis	Total		Human seroprevalence				p

			Positive		Negative		
		
	n	%	n	%	n	%	
Knowledge: professionals (workers and breeders)							
Have ever heard of brucellosis	323	100					
Yes	37	11.5	6	15.4	31	10.9	0.411^a^
No	286	88.5	33	84.6	253	89.1	
Fresh milk and unpasteurized dairy products (cheese and butter) transmit brucellosis	37	100					
Yes	10	27.0	2	33.3	8	25.8	0.704^b^
No	27	73.0	4	66.7	23	74.8	
Animal fetal materials transmit brucellosis	37	100					
Yes	9	24.3	1	16.7	8	25.8	0.633^b^
No	28	75.7	5	83.3	23	74.2	
Practice and characteristics of farms (breeders)							
Pasture is near water sources (rivers and ponds)	192	100					
Yes	76	39.6	7	21.9	69	43.1	0.029^c^
No	116	60.4	25	78.1	91	56.9	
Existence of wetlands along the pasture	192	100					
Yes	62	32.2	6	18.7	56	35.0	0.073^a^
No	130	67.8	26	81.3	104	65.0	
Replacement of herd with cattle from other pens	192	100					
Yes	179	93.2	32	100	147	91.9	0.096^c^
No	13	6.8	0	0	13	8.1	
Sale of unpasteurized milk and dairy products	192	100					0.032^c^
Yes	163	84.9	23	71.9	140	87.5	
No	29	15.1	9	28.1	20	12.5	
Abortion remains are abandoned in the pasture and eventually ingested by dogs and pigs	192	100					< 0.001^a^
Yes	91	47.4	25	78.1	66	41.3	
Does not know/No answer	61	31.8	4	12.5	57	35.6	
No	40	20.8	3	9.4	37	23.1	

^a^ Chi-square test of independence.

^b^ Chi-square test of independence with Monte Carlo Simulation.

^c^ Fisher’s exact test.

In relation to the characteristics of the farms of infected breeders, 78.1% stated that the pasture is not done along water sources (p = 0.029), identical percentage for the non-existence of wetlands along the pasture (p = 0.073). All (100%) infected breeders stated that they worked in farms that replace cattle with those from other pens (p = 0.096). Most (71.9%) of the infected breeders sold sour milk and unpasteurized dairy products to other persons (p = 0.032). In relation to breeders, 78.1% of infected breeders claimed to have left the remains of abortion in the pasture, being used, eventually, as food for dogs and pigs (p < 0.001) (Table 5).

In the multiple analysis, the logistic regression using the Forward:LR method showed that only the variable of category (*b*_categori (1)_ = 1,265; χ^2^_Wald (1)_ = 8,492; p = 0.004; OR = 3.54; 95%CI 1.57–8.30) presented statistically significant effect on the *Logit* of the probability of professionals having human brucellosis. No other variable was statistically significant after the presence of this variable in the model.

## DISCUSSION

We found weighted prevalence of brucellosis of 15.6% in the professionals under study – 5.3% in workers and 16.7% in breeders. Compared with studies conducted in 2001 in the municipalities of Bibala and Kamucuio by Doctors Without Borders, which showed prevalence in humans of 4.7%, we can observe that the prevalence values found herein are high^i^. Research studies say that, in general, the prevalence of brucellosis is unknown in sub-Saharan Africa because of the low information reported by epidemiological surveillance services. However, in some countries, the literature considers the possible existence of hyperendemia, being notified, for example in countries such as South Africa, approximately 5,000 human cases every year^16,17,28^.

The prevalence of human brucellosis in African regions with similar characteristics^13,25^ to Namibe, Angola, has great dispersion (between 1% and 13.3%), with lower values than those observed in this study. Although they are another measure of frequency, we highlight that incidence rates have even greater variability (between 0.9% and 84.3%)^12,21,24,27^. These values need to be compared carefully, as the methods used by the studies mentioned were different, especially regarding the serological tests used and the populations assessed.

In this study, we observed that brucellosis among professionals in the Namibe province predominantly affects uneducated and married cattle breeders, who began working as a minor, regardless of how they entered the activity. The same breeders dedicated themselves to the sale of unpasteurized milk and dairy products, abandoned abort remains in the pasture, and lent animals (breeding females and males) to other pens. The higher prevalence in professionals (global) and specifically in breeders were found in the South and East regions of the Namibe province, while for workers, the North region was prevalent. The non-use of personal protective equipment, start of the profession as a minor, contact with abortion remains, the consumption of fresh milk and the absence of monitoring of the production circuit, and the sale of unpasteurized milk and dairy products are mentioned by several authors as risk factors for contracting brucellosis^7,9,17^.

Infected professionals expressed ignorance of brucellosis. We need to increase the level of literacy of these professionals, especially for breeders^m,n,o^. It is essential, both in terms of Public Health and in individual terms, that professionals have knowledge on human and animal brucellosis and the respective prevention measures, as the low knowledge on this disease increases their risk of contracting it^13,23,27,29^.

The odds ratio of a breeder contracting the disease, when compared to the workers of slaughterhouse, butchers, and slaughter rooms, was 3.54 times, and for the illiterate (no instruction), when compared to those who had basic education, it was 2.25 times. In the multivariate analysis, only the professional category was significant. The results obtained allow us to assess that the factor most associated with seroprevalence of human brucellosis in livestock professionals of the Namibe province, considering the sociodemographic characteristics, was the professional category. Beheshti et al.^3^, Mukhtar and Kokab^19^, and Meky et al.^15^ have found association between seroprevalence of brucellosis and profession (breeders and workers of slaughterhouse and butchers). The prevalence in workers in this study (5.3%) was lower than that found in the work of Kumar et al.^11^, carried out in Delhi, India (12.7%). The risk of infection in livestock professionals is permanent, promoting constant planning of preventive actions^13^.

The livestock infrastructure of Namibe is precarious, with obsolete equipment and inadequate physical structures^18^. In the absence of human vaccine against brucellosis as an effective preventive measure, the use of personal and collective protective equipment by the professionals can be a crucial key to success in the prevention^10,13,23,25,c,d^.

This study had the limitations inherent in a cross-sectional observational study, by showing a photograph of the moment, hindering the establishment of cause-effect relationships, from the lack of temporal knowledge. The other limiting element was the existence of informal breeders and slaughter rooms, which are not monitored by the health surveillance system, and, therefore, limits the inference of these results for the professionals in the formal system (those registered in the Provincial Livestock Department of Namibe). In informal environments, different scenarios are expected, which can be even worse in many aspects: animal slaughter, isolation of animals in farms, compliance with practical and biosecurity measures, and the knowledge of brucellosis by the professionals.

Brucellosis is a public health problem in Namibe. This study showed that there are favorable conditions for human infection. There is a high prevalence, with variability in the professional classes (more severe in breeders than in workers) and municipalities (more prevalent in Tombwa and Virei). We identified little knowledge and few practices among the professionals. For prevention, we recommend formation and sensitizing regarding risk practices (cultural and use of personal protective equipment). There is a need for future (longitudinal) studies in the community, promotion of habits and behaviors to reduce the risk of infection, and appropriate intervention measures. We propose a concerted effort, within the concept “one health”, to prevent, control, and eradicate brucellosis, considering the individual, organizational, and governmental perspective.

## Footnotes

a Al-Nassir W, Lisgaris MV, Salata AR, Benett J. Brucellosis. In: Medscape from WebMD: Drugs & Diseases [website]. New York; 2009. Available from: http://tinyurl.com/lcl5wfw

b Mufinda FC. Conhecimento de factores de risco e de profilaxia na transmissão da brucelose humana nos profissionais da pecuária na província do Namibe, Angola, 2009 [dissertation]. Rio de Janeiro (RJ): Escola Nacional de Saúde Pública da Fundação Oswaldo Cruz; 2010.

c World Health Organization. The control of neglected zoonotic diseases: a route to poverty alleviation. Geneva: WHO; 2006 [cited 2016 Dec 4]. Available from: http://www.who.int/zoonoses/Report_Sept06.pdf

d Aubry P. Brucellose: actualités 2012. In: Medicine Tropicale [website]. Saint Denis (FR): Cours de Diplôme en Médicine Tropicale; 2012. Available from: http://tinyurl.com/oxroluc

e Elberg SS, editor. A guide to the diagnosis, treatment and prevention of human brucellosis. Geneva: Word Health Organization; 1981. Available from: http://apps.who.int/iris/bitstream/10665/66406/1/VPH_81.31_Rev.1.pdf

f Republic of South Africa, Department of Agriculture. Brucellosis. Pretoria: Department of Agriculture; 2003 [cited 2016 Dec 4]. Available from: http://www.daff.gov.za/docs/Infopaks/Brucellosis_E-book.pdf

g Angola. Governo da Província do Namibe. Plano de Desenvolvimento Económico e Social da Província do Namibe para o Período 2013-2017. Namibe; 2013. Available from: http://tinyurl.com/ptqtf86

h Ministério da Agricultura, Pecuária e Abastecimento (BR), Departamento de Defesa Animal. Programa Nacional de Controle e Erradicação da Brucelose e Tuberculose: manual técnico. Brasília (DF): Departamento de Defesa Animal; 2006. Available from: http://www.agricultura.gov.br/arq_editor/file/Aniamal/programa%20nacional%20sanidade%20brucelose/Manual%20do%20PNCEBT%20-%20Original.pdf

i Médicos Sem Fronteiras. Relatório de estudo de brucelose humana e animal nos municipios de Bibala e Kamucuio, provincia do Namibe. Namibe: Médicos sem Fronteiras – Suiça; 2001.

j Dean AG, Sullivan KM, Dean AG, Soe MM. OpenEpi: open source epidemiologic statistics for public health; version 2.3.1. Atlanta: Emory University, Rollins School of Public Health; 2012.

k OIE -World Organization for Animal Health. Terrestrial manual 2009. Paris; 2009. Bovine brucellosis: version adopted by the World Assembly of Delegates of the OIE. Chapter 2.4.3. Available from: http://web.oie.int/eng/normes/MMANUAL/2008/pdf/2.04.03_BOVINE_BRUCELL.pdf

l SPSS Inc. SPSS version 18.0. Chicago: IBM; 2010.

m World Health Organization. Brucellosis (human). Geneva; 2005. Available from: http://tinyurl.com/mq2ws6m

n Putt SNH, Shaw APM, Woods AJ, Tyler L, James AD. Veterinary epidemiology and economics in Africa: a manual for use in the design and appraisal of livestock health policy. Reading (UK): University of Reading, Veterinary Epidemiology and Economics Research Unit; 1987. (ILCA Manual, 3). Available from: http://pdf.usaid.gov/pdf_docs/PNAAW757.pdf

o USA. Office of Public Health. Infectious Disease Epidemiology Section. Brucellosis, 2008. New Orleans, LA: Infectious Disease Epidemiology Section; 2008. Available from: http://tinyurl.com/orzmh9l
